# Is IPT more effective in high-burden settings? Modelling the effect of tuberculosis incidence on IPT impact

**DOI:** 10.5588/ijtld.16.0297

**Published:** 2017-01-01

**Authors:** R. Ragonnet, J. M. Trauer, E. S. McBryde, R. M. G. J. Houben, J. T. Denholm, A. Handel, T. Sumner

**Affiliations:** * Department of Medicine, Royal Melbourne Hospital/Western Hospital, University of Melbourne, Parkville; † Centre for Population Health, Burnet Institute, Melbourne; ‡ Victorian Tuberculosis Program, Melbourne Health, Melbourne, Victoria; § Australian Institute of Tropical Health and Medicine, James Cook University, Townsville, Queensland, Australia; ¶ Department of Infectious Disease Epidemiology; # TB Modelling Group, TB Centre, and Centre for the Mathematical Modelling of Infectious Diseases, London School of Hygiene & Tropical Medicine, London, UK; ** Department of Microbiology and Immunology, University of Melbourne, Melbourne; †† Victorian Infectious Diseases Service, Royal Melbourne Hospital, Parkville, Victoria, Australia; ‡‡ Department of Epidemiology and Biostatistics, College of Public Health, University of Georgia, Athens, Georgia, USA

**Keywords:** latent tuberculous infection, preventive therapy, optimal impact

## Abstract

**SETTING:** Isoniazid preventive therapy (IPT) is effective for preventing active tuberculosis (TB), although its mechanism of action is poorly understood and the optimal disease burden for IPT use has not been defined.

**OBJECTIVE:** To describe the relationship between TB incidence and IPT effectiveness.

**METHODS:** We constructed a model of TB transmission dynamics to investigate IPT effectiveness under various epidemiological settings. The model structure was intended to be highly adaptable to uncertainty in both input parameters and the mechanism of action of IPT. To determine the optimal setting for IPT use, we identified the lowest number needed to treat (NNT) with IPT to prevent one case of active TB.

**RESULTS:** We found that the NNT as a function of TB incidence shows a ‘U-shape’, whereby IPT impact is greatest at an intermediate incidence and attenuated at both lower and higher incidence levels. This U-shape was observed over a broad range of parameter values; the optimal TB incidence was between 500 and 900 cases per 100 000 per year.

**CONCLUSIONS:** TB burden is a critical factor to consider when making decisions about communitywide implementation of IPT. We believe that the total disease burden should not preclude programmatic application of IPT.

## RESUME

**CONTEXTE :** Le traitement préventif par isoniazide (IPT) est efficace dans la prévention de la tuberculose (TB) active, même si son mécanisme d'action est mal compris et que le poids optimal de la maladie pour l'utilisation d'IPT n'a pas été défini.

**OBJECTIF :** Décrire la relation entre l'incidence de la TB et l'efficacité d'IPT.

**MÉTHODE :** Nous avons construit un modèle des dynamiques de transmission de la TB afin de déterminer l'efficacité d'IPT dans différents contextes épidémiologiques. La structure du modèle a été conçue pour s'adapter facilement à l'incertitude à la fois des paramètres d'entrée et du mécanisme d'action d'IPT. Pour déterminer le contexte optimal d'utilisation d'IPT, nous avons identifié le plus petit nombre de personnes à traiter par IPT requis (NNT) pour prévenir un cas de TB active.

**RÉSULTATS :** Nous avons trouvé que le NNT comme fonction de l'incidence de la TB suivait une courbe en U, l'impact d'IPT étant maximal pour une incidence intermédiaire et étant atténué à la fois pour un niveau d'incidence faible et élevé. Cette courbe en U a été observée pour une large fourchette de valeurs des paramètres, et l'incidence optimale de la TB a été constatée entre 500 et 900 cas par 100 000 personnes par an.

**CONCLUSION :** Le poids de la TB est un facteur crucial à envisager avant de décider d'une mise en œuvre d'IPT au niveau d'une communauté. Nous montrons que le poids total de la maladie ne devrait pas exclure l'application d'IPT par les programmes.

## RESUMEN

**MARCO DE REFERENCIA:** El tratamiento profiláctico con isoniazida (IPT) es eficaz en la prevención de la tuberculosis (TB) activa y sin embargo se conoce mal su mecanismo de acción y no se ha definido cuál es el valor óptimo de la carga de morbilidad por TB para recomendar su utilización.

**OBJETIVO:** Describir la relación entre la incidencia de TB y la eficacia del IPT.

**MÉTODO:** Se construyó un modelo de la dinámica de transmisión de la TB con el fin de investigar la eficacia del IPT en diferentes contextos epidemiológicos. Se procuró que la estructura del modelo fuese muy adaptable a la incertidumbre en las variables de entrada del modelo y el mecanismo de acción del tratamiento. Con el objeto de reconocer el contexto óptimo de aplicación del IPT, se definió el número mínimo de pacientes que es necesario tratar con el fin de prevenir un caso de TB activa.

**RESULTADOS:** Se encontró que el número de pacientes que es preciso tratar en función de la incidencia de TB, exhibe una curva ‘en forma de U’, según la cual la repercusión más alta se alcanza cuando existe una incidencia intermedia y el efecto se atenúa en los niveles de más alta y más baja incidencia. Este tipo de curva se observó en un amplio intervalo de valores y la incidencia óptima se definió entre 500 y 900 casos por 100 000 habitantes por año.

**CONCLUSIÓN:** La carga de morbilidad por TB constituye un factor esencial cuando se adoptan decisiones sobre la aplicación del tratamiento preventivo a escala de una comunidad. Los resultados del estudio revelan que la carga total de morbilidad por TB no debe excluir la aplicación programática del IPT.

TUBERCULOSIS (TB) IS A GLOBAL health problem, with 9.6 million cases and 1.5 million deaths worldwide in 2014.^1^ According to a World Health Organization (WHO) estimate, approximately one third of the world's population is latently infected with TB.^2^ However, assessment of the future risk posed by this reservoir of potential disease is challenging due to several issues, including the inability of currently available diagnostic tests to predict whether or not an infected individual will progress to active disease. Therefore, while preventive treatment against latent tuberculous infection (LTBI) may be a vital tool in achieving the WHO and the Stop TB Partnership's ambitious objective of TB elimination by 2050,^3^ the optimal setting in which to employ this intervention is uncertain.

Isoniazid preventive therapy (IPT) is known to be effective in reducing the risk of subsequent disease in LTBI patients at the individual level.^4–6^ However, its impact at the population level remains unclear. Communitywide IPT interventions in Alaska, Greenland and Tunisia have demonstrated the ability of IPT to reduce TB incidence.^5^,^7^,^8^ The number needed to treat (NNT) to avert one case of active disease was found to range between 35 and several hundred, depending on the baseline risk of TB activation, demonstrating that IPT can be very efficient, provided that relevant populations are targeted.^6^ However, the results of the recent Thibela trial conducted among South African gold miners are less clear, with no durable population-level impact demonstrated despite a reduction in the risk of TB during treatment.^4^ These observations highlight the potential for different population-level impact of IPT interventions by disease burden.

Questions have been raised around the mechanism of action of IPT, as it is unclear whether this intervention reduces the risk of later progression to active disease or cures infection.^9^,^10^ Furthermore, the ability of IPT to protect against subsequent infections has not been demonstrated, and reinfection is therefore likely to be a major modifier of IPT effectiveness, with the potential to markedly attenuate public health effects. As a direct correlation exists between TB incidence and reinfection rates,^11^ it would be logical to suppose that the success of IPT interventions will be modified by the local TB incidence.

We constructed a mathematical model that allows for variations in the TB burden and incorporated a flexible structure for exploring different assumptions regarding IPT efficacy.

## MATERIALS AND METHODS

### Model development

**Figure 1.**
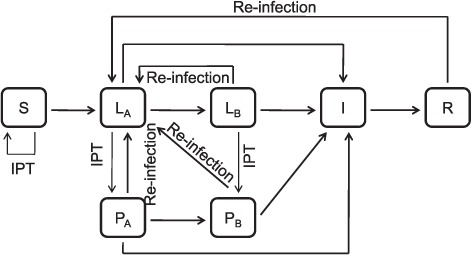
Model structure. Rectangular boxes represent the different categories in which the population is structured: susceptible (*S*), latently infected untreated (*L_A_* and *L_B_*), latently infected treated with IPT (*P_A_* and *P_B_*), infected with active tuberculosis (*I*) and recovered (*R*). Arrows represent the transitions permitted between categories. Infected individuals treated with IPT transition to corresponding compartments where the rate of disease activation is reduced. Reinfection may occur for both recovered and latently infected individuals. Birth and death flows are not represented in this diagram (Appendix). IPT = isoniazid preventive therapy.

Using ordinary differential equations and the assumption of homogeneous mixing, we created a deterministic model of TB transmission. The simplest feasible structure capable of adequately capturing both TB transmission dynamics and IPT was employed (Figure 1). Newly born individuals enter via the fully susceptible compartment (*S*). Two distinct compartments (*L_A_* and *L_B_*) were used to model LTBI to reflect the higher risk of disease progression during the early stages following infection.^12–14^ The modelled intervention was communitywide treatment for LTBI, which consists of treating infected individuals with a 9-month course of IPT after infection is detected using the tuberculin skin test (TST) or interferon-gamma release assays (IGRAs) (Appendix).^*^

Individuals with LTBI treated with IPT transition to two equivalent compartments *P_A_* and *P_B_*. In these compartments, we assumed a reduced risk of progression to disease compared to that existing before IPT commencement. This model structure allows the exploration of a wide range of possibilities regarding the effectiveness and mechanism of action of IPT; i.e., different levels of reduction in the risk of progression achieved through IPT may be considered, as well as a situation where IPT can completely cure infection. Infected individuals developing active TB progress to compartment *I*, and eventually transition to compartment *R* in case of recovery. In our model, all individuals with a history of tuberculous infection can be re-infected.^15^,^16^ Various assumptions concerning the risk of reinfection are considered: some degree of immunity may be conferred by previous infection, although non-biological factors such as social mixing patterns could enhance the risk of reinfection.

The Table presents the main assumptions made in our model; a detailed description of the model and the associated differential equations are available in the Appendix.

**Table:**
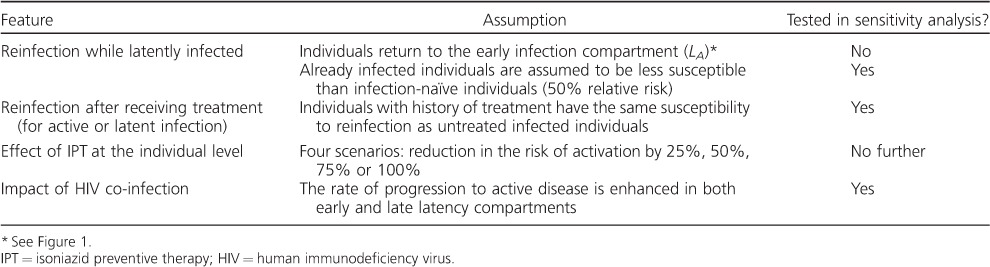
Main assumptions

### Observed model outputs

Disease burden indicators, including incidence, prevalence and mortality, are recorded before the intervention starts and over 10 years of IPT implementation. From these measures, three different indicators are calculated and reported: 1) the primary outcome, which is the NNT required to avert one case of active TB; 2) the proportional reduction in incidence of active TB; and 3) the absolute number of active TB cases averted in the population through IPT. These three outputs allow us to consider both the absolute and relative impact of treatment, as well as the NNT, which is our primary consideration as it describes the population-level effectiveness of the strategy per treatment provided. NNT is defined as the number of active TB cases averted over 10 years of intervention divided by the total number of individuals treated with IPT. The optimal incidence is then obtained by minimising this indicator.

We also estimate the proportion of disease due to early progression vs. late reactivation, as well as the risk of reinfection in the different incidence settings, as these factors are expected to play an important role in IPT efficiency.

### Sensitivity analyses

A sensitivity analysis was performed to observe whether the optimal incidence for implementing IPT is modified by alternative parameter set selections. First, we consider one-dimensional variations in each parameter across the ranges presented in Appendix Table A. Next, considering the same numeric ranges, we performed a multidimensional sensitivity analysis employing a Latin hypercube method to obtain 1000 parameter sets. Finally, we considered additional scenarios where model parameterisation was adjusted to simulate human immunodeficiency virus (HIV) endemic settings, considering various levels of HIV prevalence as well as different assumptions regarding the effect that HIV infection has on the risk of TB disease activation.

### Model implementation

The model was implemented in R, version v3.1.2 (R Computing, Vienna, Austria) and the code to reproduce all the results presented here is supplied in the Appendix.

### Ethics approval

Ethics approval was not required for the study, as no patients were involved.

## RESULTS

### Baseline results

**Figure 2.**
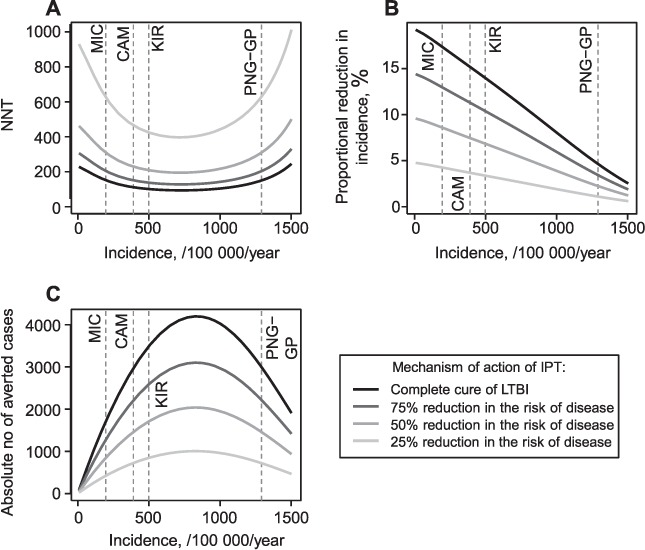
Baseline results. Three indicators of (IPT) effectiveness are presented. **A)** The NNT to avert one case of active tuberculosis; **B)** the proportional reduction in incidence; and **C)** the absolute number of cases averted due to IPT. All three indicators are calculated over a period of 10 years of intervention. In each panel, four curves are presented corresponding to different assumptions regarding the IPT efficacy. The vertical dashed lines represent four countries/regions that illustrate different levels of TB incidence: Micronesia (MIC), Cambodia (CAM), Kiribati (KIR) and the Gulf Province of Papua New Guinea (PNG-GP). NNT = number needed to treat; IPT = isoniazid preventive therapy; LTBI = latent tuberculous infection; TB = tuberculosis.

Figure 2 shows the outcome measures for IPT effectiveness as a function of TB incidence, with different efficacy levels for IPT. Four examples of countries/region are represented in Figure 2 to illustrate different levels of TB incidence: Micronesia (MIC), Cambodia (CAM), Kiribati (KIR) and the Gulf Province of Papua New Guinea (PNG-GP), with estimated TB incidences of respectively 195, 390, 497 and 1290 cases per 100 000 population per year.^1^,^17^ As our baseline analysis does not apply to HIV-endemic settings, only settings with low HIV prevalence are presented.

We observed a U-shaped curve for the NNT to avert one case of active TB regardless of efficacy (Figure 2A); i.e., NNT is lowest at an intermediate incidence (500–900 cases/100 000/year), but increases in both lower and higher incidence settings. As would be intuitively expected, NNT values are lower under the most optimistic assumptions (i.e., complete cure or strong protection provided by IPT). However, the optimal TB incidence for implementing IPT is relatively unaffected by different values for the proportion cured by IPT assumptions, and ranges between 717 and 726 cases/100 000/year. Both the U-shaped curve and the location of the optimum are conserved. The corresponding values of optimal NNT range from 94 to 396.

In contrast, the proportional reduction in TB incidence decreases with background incidence (Figure 2B). While IPT produces a significant reduction in TB incidence in low-to-moderate burden settings (5–19% reduction in 10 years for an incidence of 50 cases/100 000/year), its impact in very highly endemic settings is small (1–3% reduction in 10 years for an incidence of 1500 cases/100 000/year), although in high-burden settings even a slight reduction in incidence results in a significant absolute number of cases averted.

Finally, the absolute number of averted cases reveals another non-monotonic relationship with TB incidence, regardless of the assumption made about IPT efficacy (Figure 2C), with a similar (although inverse) pattern to that seen for NNT. The maximal number of averted cases is obtained when TB incidence is between 827 and 835 cases/100 000/year. At this incidence, and in a total population of 1 000 000, the model predicts that IPT would prevent between 1006 (if IPT reduces risk of activation by 25%) and 4199 (if IPT cures LTBI) cumulative active TB cases over 10 years of intervention.

### Two interacting phenomena

**Figure 3.**
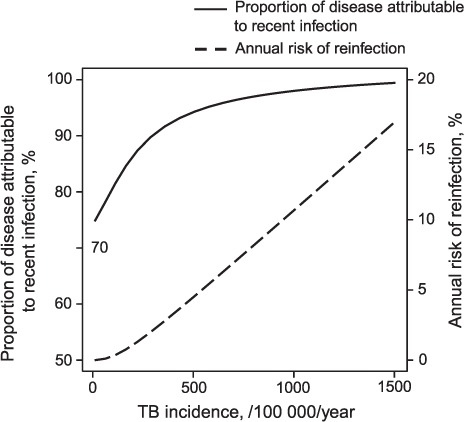
Annual risk of reinfection and the proportion of disease due to recent infection as functions of TB incidence. ––– =proportion of TB disease due to recent infection; - - - = annual risk of reinfection. TB = tuberculosis.

Figure 3 shows two measures to quantify the contribution of two phenomena suspected to explain the dynamics driving the U-shape: 1) the proportion of TB incidence attributable to recently infected individuals; and 2) the annual risk of reinfection for individuals with LTBI. At less extreme incidence rates (0–500), the picture is dominated by the rapid increase in the proportion of disease due to recent infection, explaining the decrease in NNT over this range of incidence (Figure 2A). In contrast, when incidence reaches very high levels (>1000), the proportion of disease due to recent infection increases more slowly as it approaches its saturation level of 100%. At the same time, reinfection continues to increase linearly with incidence, and dominates the picture over this range. Accordingly, at such high incidence levels, the NNT increases with incidence.

### Sensitivity analyses

**Figure A.2:**
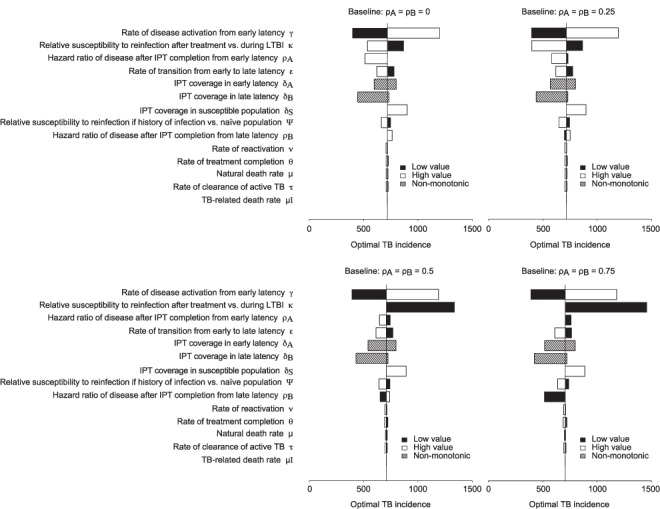
One-way sensitivity analyses realised under different assumptions regarding the mechanism of action of IPT. The ranges considered for the parameter values are presented in the main text (Table A). Values on the *x*-axis represent the optimal TB incidence obtained by minimising the number needed to treat. The vertical line indicates the result obtained with the baseline parameter set (Table A; main text) under the different assumptions regarding the mechanism of action of IPT. Black (vs. white) rectangles correspond to parameter values that are lower (vs. higher) than the corresponding baseline value. The hatched rectangles indicate situations where the variation of the optimal incidence over the parameter range is not monotonic. LTBI=latent tuberculous infection; IPT=isoniazid preventive therapy; TB=tuberculosis.

Appendix Figure A.2 presents the results of the sensitivity analysis performed to observe the impact of single variations in parameter values on the optimal incidence obtained by minimising the NNT. The sensitivity analysis highlights that a faster rate of progression from early latency to active disease results in a higher estimate of the optimal incidence.

Another parameter with a marked impact on our conclusions is the risk of reinfection. Specifically, we find that the risk of reinfection after treatment by comparison with the risk during LTBI plays a major role in determining the incidence that correlates with optimal IPT impact. In particular, if we assume that susceptibility to reinfection is enhanced after treatment completion, the nadir of the NNT U-shape occurs at a lower TB incidence. In contrast, when the risks of reinfection before and after treatment are varied together, we observe minimal impact on optimal incidence. Single variations in other parameters have no pronounced effect on optimal incidence.

**Figure A.3:**
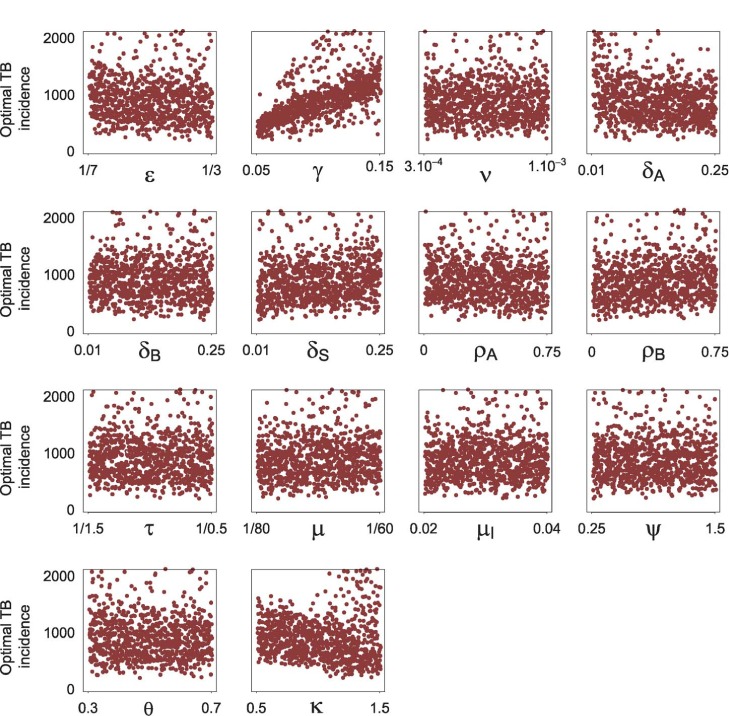
Representation of the multidimensional sensitivity analysis. A Latin hypercube method was used to create 1000 parameter sets. The values obtained for the optimal incidence over the different ranges of parameters are represented. Parameter notations and definitions are described in Table A. TB = tuberculosis.

Appendix Figure A.3 presents the results of the multidimensional sensitivity analysis using a Latin hypercube method for sampling 1000 parameter sets. We measured a median optimal incidence of 811 cases/100 000/year (interquartile range 582–1066). We noted that 100% of the runs led to a strictly positive value for optimal incidence, and that its lowest value was 154 cases/100 000/year, indicating that the U-shape was conserved over all model runs. This multivariate sensitivity analysis confirms the results of the previous analysis, showing that only variations in the rate of progression from early latency to active disease and the risk of reinfection after treatment by comparison to the risk during LTBI significantly impact the results.

Finally, our analysis relating to HIV-endemic settings revealed that the findings concerning the U-shape curve associated with a high level of optimal TB incidence for IPT use were not jeopardised, even when considering a very high HIV prevalence (Appendix, Consideration of HIV-endemic settings). We found that the optimal TB incidence increases with HIV prevalence and that IPT use becomes more efficient (NNT reduced) in HIV-endemic settings, reaching NNT levels as low as 14 when TB incidence is 3767 (optimal configuration) and for an HIV prevalence of 26%. These findings remained valid under various scenarios concerning the effect of HIV infection on the risk of TB activation.

## DISCUSSION

We find that the optimal epidemiological settings for the programmatic use of IPT against LTBI occur at surprisingly high levels of TB incidence. The NNT initially falls as TB incidence increases, and then follows a U-shaped curve, with the maximal impact of IPT found at a TB incidence of around 720 cases/100 000/year. This finding remained valid regardless of the assumptions made about IPT efficacy, from assuming a weak reduction (25%) in the risk of disease progression through to allowing complete cure of infection. This consideration of different scenarios is of particular importance given the uncertainty around the individual-level effect of IPT. This concern was approached in a recent modelling study, and it was found that IPT is unlikely to totally cure infection in HIV-positive individuals not on antiretroviral therapy.^10^ However, no similar investigations have been conducted in the general population. Our different sensitivity analyses provide confidence in the U-shape finding, as the exploration of a wide parameter space did not affect this qualitative result even when considering HIV-endemic settings.

Such optimal levels of incidence might seem very high when considering country-specific estimates, as only South Africa, Lesotho and Swaziland exceeded annual incidences of 700 new TB cases/100 000 in 2014, and HIV is a critical driver of the huge disease burden in these settings.^1^ However, a similar TB burden might also occur in more moderate HIV burden settings, when considering smaller subnational populations or local communities such as PNG-GP within countries of much lower national incidence.^17^ Our results suggest that it is only when incidence reaches extremely high levels (>1400 cases/100 000/year) that the effects of IPT begin to attenuate. In these settings, IPT would result in a limited reduction of incidence and few averted cases, leading to unreasonable NNTs.

We propose an explanation for the U-shaped curve by the interaction of two competing phenomena that vary in intensity as incidence increases: the rise in the level of reinfection and the rise in the proportion of disease that is due to recent infection. On the one hand, higher incidence leads to higher risks of reinfection in both recovered and latently infected individuals. Patients who have been treated for LTBI are thus more likely to be re-infected and consequently have a high risk of active TB in the early phase of this new infection. Accordingly, the benefits from IPT diminish with higher incidence. On the other hand, in high-incidence settings, the proportion of LTBI cases recently infected is greater than in lower-incidence settings. The risk represented by the LTBI reservoir is thus higher, given that early infections have the highest risk of progression to active disease, leading to a greater benefit from IPT in higher-burden settings. This profile was demonstrated previously, and is confirmed by the findings of our simulations.^11^,^18^,^19^

We further demonstrate that the risk of reinfection plays an important role in the estimation of optimal incidence, finding that it is crucial to distinguish susceptibility to reinfection for latently infected individuals from that occurring after treatment. Our results imply that if isoniazid attenuates the immunity conferred by previous infection, the corresponding optimal incidence may be much lower, particularly if rates of progression following recent infection are low. Unfortunately, little is known about the true effect of IPT on acquired immunity and, accordingly, our study indicates that further work that would allow us to distinguish between these two risks of reinfection would bring crucial knowledge to better understand the potential impact of IPT.

Our sensitivity analysis emphasises the importance of detailed knowledge of the dynamics of TB latency, and especially of its early stages. Fortunately, several studies have now reported the rate of disease progression from recent infection, generating consistent estimates.^12–14^,^20^ Factors such as HIV infection or young age at infection have been shown to increase the risk of TB disease progression,^14^,^20^,^21^ which—according to our model—may lead to higher optimal incidences. Our additional analysis focusing on HIV-endemic settings confirmed this assumption, and also suggested that it becomes more efficient to use IPT when HIV is endemic, due to the higher potential of TB disease represented by the infection reservoir. Future modelling investigations based on this work, but incorporating a more specific model structure, could be conducted to provide stronger evidence regarding HIV-endemic settings. Although age structures along with non-homogeneous population mixing could also be incorporated in future works to enhance the realism of the model in local contexts, such features were not considered in this exercise, as we aimed to provide broad insights to minimise the complexity of our model.

A further limitation of our study is that we did not consider multidrug-resistant TB (MDR-TB) settings. If we assume that specific MDR LTBI regimens have similar effectiveness to that of IPT for drug-susceptible LTBI, as suggested by recent observational studies,^22^ our results could be extended to high MDR-TB settings. Nevertheless, although our model is potentially applicable to high MDR-TB burden settings, the diagnosis of MDR LTBI is much more complicated and often assumed on the basis of contact history, making our model too limited to fully understand these considerations. Finally, potential side effects as well as cost of IPT were not considered in this study, as we aimed to observe the impact on the TB epidemic. While further works could help to better understand these aspects, our choice of NNT as the primary outcome allows implicit consideration of the costs and risks involved in this intervention, alongside its benefits.

## CONCLUSIONS

While the WHO recommends mostly using IPT in low-endemic settings,^23^ our study suggests that the optimal TB incidence for employing IPT is considerably higher than expected, indicating that total burden of disease should not preclude the programmatic application of IPT. In the light of the ambitious new End TB global targets for the post-2015 era, bold new strategies will be required, potentially incorporating preventive treatment. While our results were robust to most model inputs, better understanding of post-treatment immunity is critical to refining our estimates.

## Figures and Tables

**Figure A.1.**
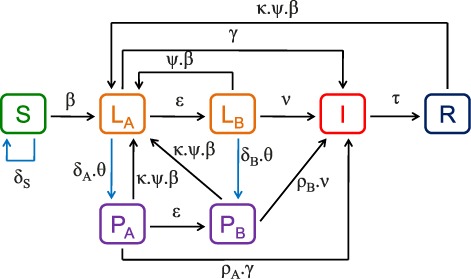
Model structure and parameters. Rectangular boxes represent the different categories in which the population is structured. Arrows represent the transitions permitted between categories. Blue arrows indicate flows related to IPT. Infected individuals treated with IPT transition to corresponding compartments where the rate of disease activation is reduced (multipliers ρ*_A_* and ρ*_B_*). Reinfection may occur for both recovered and latently infected individuals. Treated individuals have a reduced risk of reinfection (κ) compared to treatment-naïve latently infected individuals. Birth and death flows not represented in this diagram (see main text for full description). IPT=isoniazid preventive therapy.

**Table A:**
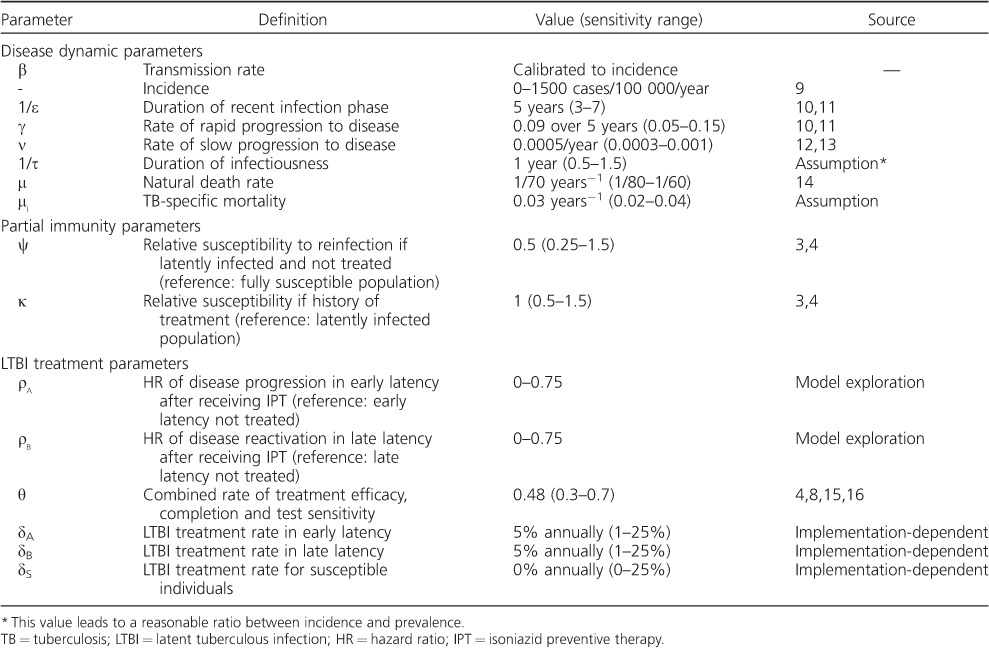
Model parameters and parameter values. The figures in brackets indicate the ranges used for the sensitivity analysis

**Figure A.4:**
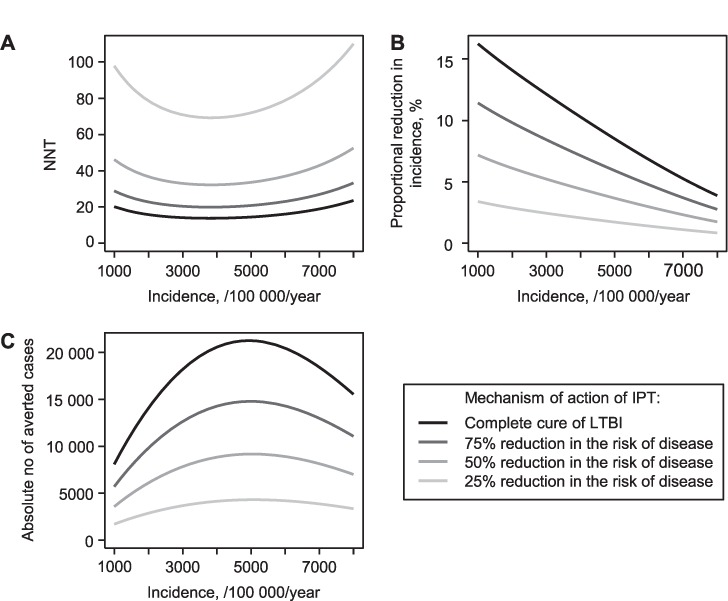
Results associated with a high HIV-endemic setting. NNT = number needed to treat; IPT = isoniazid preventive therapy; LTBI = latent tuberculous infection; HIV = human immunodeficiency virus.

**Figure A.5:**
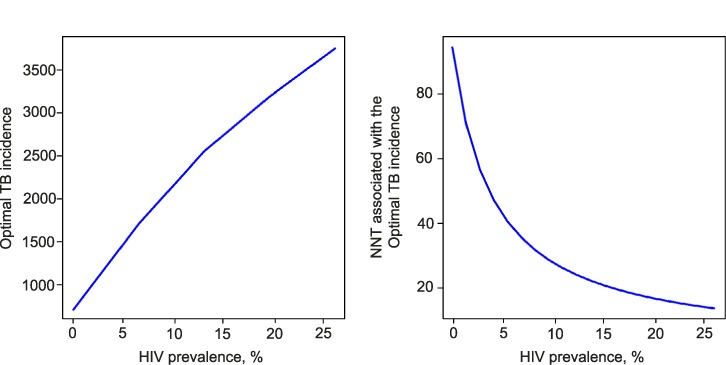
Optimal TB incidence and associated NNT over HIV prevalence. TB = tuberculosis; HIV = human immunodeficiency virus; NNT = number needed to treat.

**Figure A.6:**
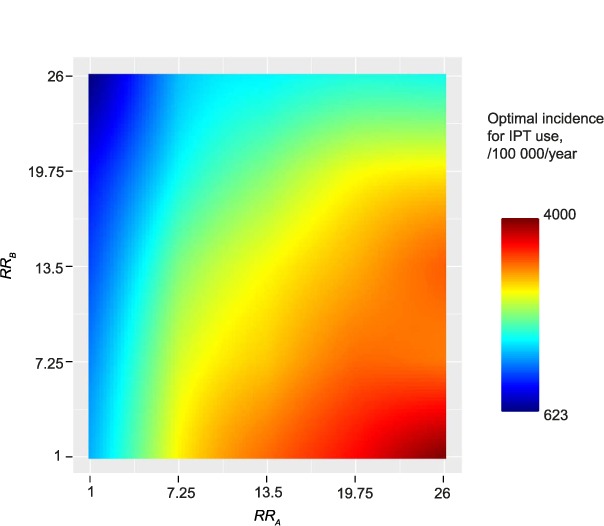
Optimal TB incidence obtained under various scenarios regarding the RR of TB among HIV-infected individuals compared to that among non-HIV-infected individuals. The bottom-left corner point (*RR_A_* = *RR_B_* = 1) corresponds to our baseline situation in absence of HIV infection. The top-right corner point (*RR_A_* = *RR_B_* = 26) corresponds to the situation studied previously, with detailed results reported on Figure A.4. *RR_A_* = the RR that applies to the early latency compartment (*L_A_*); *RR_B_* = the RR that applies to the late latency compartment (*L_B_*); IPT = isoniazid preventive therapy; RR = relative risk; TB = tuberculosis; HIV = human immunodeficiency virus.

## Footnotes

^*^ The appendix is available in the online version of this article, at http://www.ingentaconnect.com/content/iuatld/ijtld/2017/00000021/00000001/art00012

## APPENDIX

### Description of the mathematical model

Figure A.1 presents the model structure along with parameters. A natural mortality rate (**μ**) applies to every compartment; an additional tuberculosis (TB) specific mortality rate (**μ***_I_*) is added to the active disease population (*I*). Recruitment is set equal to total mortality to maintain a constant population size. Newly born individuals enter via the fully susceptible compartment (*S*). As described in the main text, two compartments (*L_A_* and *L_B_*) are used to model untreated latent tuberculous infection (LTBI), while two additional compartments (*P_A_* and *P_B_*) are used to represent LTBI treated with isoniazid preventive therapy (IPT). In these compartments, we assume a reduced risk of progression to disease compared to that existing before IPT commencement by using the multipliers **ρ***_A_* and **ρ***_B_*. In this way, different levels of reduction in the risk of progression achieved through IPT may be considered, as well as a situation where IPT completely cures infection (**ρ***_A_* = **ρ***_B_* = 0). Furthermore, this structure allows for different efficacies of IPT according to whether the infection is acquired recently or remotely (by setting **ρ***_A_***≠****ρ***_B_*).

In our model, all individuals with a history of tuberculous infection can be re-infected,^1^,^2^ although we systematically varied the rate at which reinfection occurs. In the base-case, we assumed that prior (or current) tuberculous infection confers 50% immunity against subsequent infection (**ψ** = 0.5), in accordance with previous estimates.^3^,^4^ Even if previous infection is assumed to provide some protection during an infectious contact, it should be noted that reinfection is driven not only by biological factors, but also by social mixing patterns, as previously infected individuals are more likely to live in settings with higher exposure to TB. Accordingly, we chose to consider a wide range of values for the risk of reinfection, even considering situations in which risk is augmented by previous infection (**ψ**>1), as suggested by Verver et al.^5^ The effect of treatment on subsequent risk of tuberculous reinfection is unclear. Treatment may provide some biological protection, reducing the risk of reinfection, or treatment could reduce the degree of immunity conferred by infection, increasing the risk of reinfection. Thus, although we assumed an equivalent risk of reinfection for any history of infection (treated or not) in the base-case (**κ** = 1), we explored alternative configurations in sensitivity analyses (**κ** varied from 0.5 to 1.5). In earlier model iterations, we found that the critical issue in susceptibility to reinfection was the relative susceptibility of treated (either for LTBI or TB) vs. untreated individuals. We therefore chose to use the multiplier **κ** to represent relative susceptibility after treatment by comparison with treatment-naïve individuals who are latently infected.

The simulation was realised in two stages. First, we assumed no IPT coverage and ran the model to equilibrium. From this equilibrium state, we then ran the model over 10 years of IPT implementation. Treatment rates (**δ***_A_*, **δ***_B_*, and **δ***_S_*, which represent annual coverage) remain constant during this second phase and apply to the whole population of the corresponding category (*L_A_*, *L_B_*,and *S*, respectively), but may differ according to the stage of latency (i.e., **δ***_A_***≠****δ***_B_*). These differences permit consideration of higher IPT coverage among recently infected cases, to reflect a more targeted intervention, such as case finding among TB contacts. Furthermore, given the difficulties encountered with LTBI diagnosis—and particularly given the poor specificity of the tuberculin skin test (TST) which is predominantly used—we allow for IPT to be given to a proportion of non-infected individuals.^6^,^7^ In the sensitivity analysis, we thus allow for IPT use in the fully susceptible population (**δ***_S_*>0), although treatment of this population is assumed to be ineffective and would therefore have no epidemiological impact. We consider relatively low treatment coverage in the infected population to account for the fact that treatment would not be initiated in all infected individuals in the real world, as some of them may not complete testing or, even if diagnosed, may not start treatment. We assumed null IPT coverage for individuals who have already successfully completed a treatment course for LTBI (in compartments *P_A_* and *P_B_*).

At baseline, we assume a combined rate of efficacy, completion and sensitivity of LTBI detection of 48%. This results from the multiplication of a test sensitivity (for TST or interferon-gamma release assays) of around 80%,^8^ and a combined rate of adherence and treatment efficacy of 60%. This latter figure was obtained from the Cochrane review of Smieja et al. reporting a risk ratio of 0.40 of developing TB when receiving isoniazid for 6–12 months.^4^ The definitions and values of the model parameters are presented in Table A.

### One-way sensitivity analyses under different assumptions regarding the mechanism of action of IPT

Figure A.2 represents the results of one-way sensitivity analyses realised under different assumptions regarding the mechanism of action of IPT (i.e., different values of **ρ***_A_* and **ρ***_B_*). The upper left panel corresponds to the analysis presented in the main text.

### Multidimensional sensitivity analysis

Figure A.3 is a scatter plot representing the results of the fully varied sensitivity analysis. Each of the 14 panels corresponds to the same sampling of 1000 parameter sets, but the different parameters are represented on the *x*-axis.

### Consideration of HIV-endemic settings

To observe how the model predictions would be affected by considering human immunodeficiency virus (HIV) endemic settings, we undertook analyses with parameter values to simulate populations with HIV prevalence. Specifically, we assumed that the risk of progression to active TB disease is higher for this population during both the early and late stage of LTBI. In particular, this corresponds to an increase in the values of the parameters γ and ν (see Table A for descriptions). These parameters are then recalculated by using the HIV prevalence (*Prev_HIV_*) and the relative risk (RR) of activation among HIV-infected individuals compared to that among non-HIV-infected individuals (*RR*), as follows:

where **ν***_HIV_* and **γ***_HIV_* denote the risks of progression to active disease for an HIV-endemic setting. The *RR* is known to be around 26 according to the World Health Organization.^17^ Figure A.4 presents the results of our analysis when we assume an *RR* of 26 and for a very high HIV prevalence of 26%, which corresponds to that in the country with the highest HIV prevalence in the world: Swaziland.

We observe the same U-shape phenomena as that reported in the absence of HIV infection. However, the model suggests that the TB incidence corresponding to the optimal use of IPT is much higher when HIV is endemic, reaching 3767 new cases per 100 000 population per year when considering a high HIV prevalence of 26%. We also note that the associated number needed to treat (NNT) is much lower than in the absence of HIV infection, suggesting that it becomes more efficient to use IPT when HIV is endemic than in the absence of HIV. This result is in agreement with previous findings that show that the NNT for IPT is lower when the baseline risk of TB is higher.^4^ We then considered various levels of HIV prevalence and observed the associated optimal TB incidences for IPT use, as well as the corresponding NNT (Figure A.5). This analysis confirms the previous finding that the optimal TB incidence for IPT use increases with HIV prevalence while the associated NNT decreases.

Finally, we wanted to explore alternative scenarios regarding the RR of activation among HIV-infected individuals compared to that among non-HIV-infected individuals (*RR*), by considering different RRs for the early and late latency compartments. While it was shown that HIV infection increases the risk of TB activation after recent infection,^18^,^19^ as well as the risk of late reactivation,^20^,^21^ it remains unclear whether these two risks are affected in the same way. Accordingly, we performed an additional analysis where different RRs apply to the early and late latency compartments.

Figure A.6 presents the optimal TB incidence obtained when the RRs in early latency and that in late latency are varied separately (between 1 and 26) and when considering an HIV prevalence of 26%.We observe that the RR that applies to the early latency has a much higher impact than that applying to the late latency compartment. This suggests that the impact of HIV infection on the efficiency of IPT is mostly caused by the fact that HIV-infected individuals present a higher risk of TB activation after recent infection.
